# Clinical outcome of adjuvant endocrine treatment according to Her-2/neu status in breast cancer

**Published:** 2011-01

**Authors:** Rani James, K. Thriveni, Lakshmi Krishnamoorthy, Vijayalaxmi Deshmane, P.P. Bapsy, Girija Ramaswamy

**Affiliations:** Department of Biochemistry, Kidwai Memorial Institute of Oncology, Bangalore, India; * Department of Surgical Oncology, Kidwai Memorial Institute of Oncology, Bangalore, India; ** Department of Medical Oncology, Kidwai Memorial Institute of Oncology, Bangalore, India

**Keywords:** Breast cancer, ELISA, estrogen receptor, Her-2/neu, tamoxifen

## Abstract

**Background & objectives:**

An association between over-expression of proto-oncogene Her-2/neu and resistance to tamoxifen in estrogen receptor (ER) positive, primary and metastatic breast cancer has been suggested. HR+/Her-2/neu+ patients have a poor response to endocrine therapy, making this group a matter of debate. The present study was carried out to examin whether Her-2/neu expression in breast cancer patients predicted tamoxifen effectiveness.

**Methods:**

An enzyme-linked immunosorbent assay (ELISA) specific for the extracellular domain of the Her-2/neuoncoprotein product was used to detect serum Her-2/neu levels in 207 patients with histological confirmed breast cancer. Tissue Her-2/neu expression was studied in 100 breast cancer patients by immunohistochemistry (IHC) and compared with serum Her-2/neu levels by ELISA.

**Results:**

Among 207 histologically confirmed breast cancer patients, 53 were serum Her-2/neu positive. Patients who were treated with surgery, chemotherapy, and radiotherapy showed significantly (*P*<0.05) reduced serum Her-2/neu levels, showing good response to treatment. Patients who were treated with tamoxifen in addition to the above regimen did not show any significant reduction in serum Her-2/neu levels showing resistance to treatment.

**Interpretation & conclusions:**

The present findings study support the hypothesis that Her-2/neu overexpression contributes to tamoxifen resistance. Trastuzumab or other growth factor inhibitors should be used in combination with tamoxifen, since monotherapy is not likely to be optimal in HR+/Her-2/neu+ tumours.

Breast cancer development and progression involves complex interactions between hormonal receptors and growth factor signaling pathways^1^. The Her-2/neu oncogene is overexpressed in 25-30 per cent cases of breast cancers^2^. Overexpression of the human epidermal growth factor receptor-2 (Her-2) gene, a breast cancer marker, is associated with rapid tumour growth, increased risk of recurrence after surgery, poor response to conventional chemotherapy and shortened survival^3^.

The Her-2/neu protein is an epidermal growth factor receptor-like protein having a molecular weight of 185,000 dalton protein with extracellular, transmembrane, and tyrosine kinase domains^4^. The extracellular domain of the receptor protein can be cleaved from the cell surface by matrix metalloproteases and then released into blood^5^where it can be detected by ELISA in up to 45 per cent cases of advanced breast cancer^6^. Several techniques are available for the genetic testing of Her-2/neu amplification^7^. Currently approved methods for Her-2/neu testing include immunohistochemistry or fluorescent *in situ* hybridization using tumour tissue. A fragment of Her-2 composed of its extracellular domain can also be detected in the serum of some patients with breast cancer^8^.

There is some evidence that Her-2/neu status is a predictor for response/resistance to specific chemotherapeutic agents. Transfection of the Her-2 gene to achieve amplification in ER positive human breast cancer cells also results in acquisition of estrogen-independent growth that is resistant to therapy with the anti-estrogen tamoxifen^9^. Tamoxifen, a selective estrogen receptor (ER) modulator, is the most used drug for the treatment of ER-positive breast cancer. Adjuvant therapy studies of tamoxifen show a 40 to 50 per cent reduction in the odds of recurrence and reduced mortality^10^. Despite its benefit in patients with all stages of ER-positive breast cancer, the major obstacle to its use is treatment resistance, which either occurs *de novo* or is later acquired after initial benefit. The underlying mechanisms for tamoxifen resistance are probably multifactorial but remain largely unknown^11^. There are several potential causes for resistance to tamoxifen. Both pre-clinical and clinical studies suggest that one such mechanism involves cross talk between ER and growth factor and/or stress kinase signaling pathways^12^.

Several studies showed that Her-2/neu overexpression is associated with hormone resistance^13^,^15^. Patients with ER+/Her-2/neu+ metastatic breast cancer are less likely to respond to hormone treatment than ER+/Her-2/neu - patients. Petersons *et al*^13^ reported that Her-2/neu overexpression was associated with tamoxifen resistance. Another study by Leitzel *et al*^14^ have found that the majority of patients with estrogen receptor-positive breast cancer initially respond to hormone therapy, but eventually develop resistance. Patients with ER+/c-erbB-2+ metastatic breast cancer are less likely to respond to hormone treatment than ER+/c-erbB-2- patients^15^. All these studies suggest that Her-2/neu overexpression is associated with hormone resistance, whereas other studies have found no such association^16^,^17^.

Although many patients benefit from tamoxifen in the adjuvant and metastatic settings, resistance is an important clinical problem. The present study examined whether Her-2/neu expression in patients with breast cancer predicted response to tamoxifen therapy.

## Material & Methods

*Patient population*: This is a prospective study which included histologically confirmed consecutive cases of breast cancer (n= 207) registered (2004 to 2006) in the Breast Service Unit of Kidwai Memorial Institute of Oncology, Bangalore, and breast tissue samples were collected. Age, disease stage, grade, nodal status, estrogen receptor (ER) and progesterone receptor (PR) status were noted from the case files. Age matched (±2 yr) healthy female controls (175 cases) were selected from patient relatives.

Blood samples (5 ml) were collected in plain tubes, centrifuged to separate the serum. Serum samples were stored at -20 °C until analysis (a month) by ELISA^18^. Only those patients showing Her-2/neu positivity were selected for follow up study. Blood samples were collected from study patients at intervals of three months, six months and one year from the commencement of treatment {surgery followed by chemotherapy (CT) and/or radiotherapy (RT)} for measurement of Her-2/neu levels. Immunohistochemistry (IHC)^19^ study was carried out for 100 patients to find the correlation between serum Her-2/neu and tissue Her-2/neu expression. IHC on frozen sections has shown substantial correlation with Her-2/neu gene based assays^20^. Thus, ELISA and IHC were used for the detection of Her-2/neu protein.

The study protocol was approved by the Ethics committee of Kidwai Memorial Institute of Oncology, Bangalore. A written informed consent was obtained from each patient.

### 

#### Methods:

*Immunohistochemistry* - Overexpression of Her-2/neu protein was detected by IHC using rabbit monoclonal antibody, (Labvision, UK) which targets the extracellular domain of p185^Her-2/neu^. Ultrathin sections (4 µ) were cut from formalin fixed paraffin embedded tissue blocks, float mounted on adhesive coated glass slides, deparaffinized in xylene and ethanol. Sections were quenched with fresh 3 per cent hydrogen peroxide block to inhibit endogenous tissue peroxidase activity for 5 min and rinsed with deionised water. Sections were then boiled in 100 ml antigen retrieval citrate buffer (0.01M), pH 6.0 for 40 min. The slides were allowed to cool for 20 min and subsequently rinsed thoroughly in deionised water and then with Tris buffer. Sections were incubated in unlabelled blocking serum solution for five to ten min and then incubated for 1 h with primary antibody at a dilution of 1:100 in buffer. Subsequently, sections were washed in Tris buffer, and then incubated first with biotinylated secondary antibody solution for 30 min, washed with Tris buffer and again incubated with horseradish peroxidase (HRP) conjugated streptavidin-biotin complex for 30 min. Subsequently colour was developed using diaminobenzidine. Sections were then counterstained with haematoxylin and were mounted. Only formalin- fixed samples were used for the study.

Immunostained slides were examined by light microscopy. For each batch of experiment, formalin fixed paraffin-embedded cell block (which was previously shown to overexpress Her-2/neu) was taken as positive control and a negative control (without adding primary antibody) was included. A sample was judged to be positive when distinct membrane staining of tumour cells was observed and visually compared with no staining on the surrounding normal epithelia and no staining in the negative control cells. Each specimen was scored semiquantitatively as to the intensity of membrane immunostaining on a four point scale, with 0 indicating absence of staining, 1+ indicating non homogenous weak staining (<10% of membrane staining). To qualify for 2+ and 3+ scoring, complete membrane staining of more than 10 per cent of tumour cells was required. Scores of 0 or 1+ were considered negative for Her-2 overexpression; scores of 2+ were considered weakly positive; and scores of 3+ were considered strongly positive^21^.

ELISA - Serum samples collected from patients and controls were used for determination of serum Her-2/neu by sandwich enzyme immunoassay kit (Bender Med Systems, USA).

Cut-off value used in this study was 15 ng/ml as per kit recommendation. The observed range in control individuals for Her-2/neu was 3.5-13.5 ng/ml which was found to match the cut-off range as per recommendation of the manufacturer.

*Statistical analysis*: Parametric F test was used to calculate stage wise significance. Chi square test was used to compare age, menopausal and nodal status, stages, ER PR status with serum Her-2/neu levels. Spearman rank correlation test was used to calculate correlation between serum Her-2/neu levels and hormone receptor status. P<0.05 was considered statistically significant. Mc Nemar test was used to calculate the significance between serum Her-2/neu levels and tissue Her-2/neu expression.

## Results

Majority preponderance of the patients (40%) were in the age group 40 to 55 yr and received no prior adjuvant therapy. Among 207 histologically confirmed breast cancer patients, 53 patients were serum Her-2/neu positive (25%). Majority (n=106) were in stage III at the time of diagnosis. Patients with tumour size >5 cm (n=119), showed significantly (*P*<0.005) elevated levels of serum Her-2/neu (19.29 ± 47.4 ng/ml) compared to patients with smaller tumour size. Serum levels of Her-2/neu in patients having tumor size between 2 to 5 cm (n=52) were 14.69 ± 23.18 ng/ml and for tumour size <2 cm (n=36) was 12.69 ± 8.1 ng/ml. Our study showed that increased serum Her-2/neu levels were associated with clinical stage of disease and hormone receptor status and not associated with age, menopausal status and lymph nodal status. The pretheraputic serum levels of Her-2/neu were significantly elevated in patients with large tumour size (*P*<0.005) and showed a significant inverse correlation between serum Her-2/neu and hormone receptor status (r=-0.45, P<0.05).

To study the level of serum Her-2/neu in tamoxifen treated patients we studied follow up cases after 3, 6 months and 1 year from the commencement of treatment (Table I). It was found that patients who were treated with surgery+CT+RT showed significantly (*P*<0.05) reduced serum Her-2/neu levels, showing good response to treatment. But those who were treated with tamoxifen in addition to the above regimen did not show any significant reduction in serum Her-2/neu levels showing resistance to treatment.

**Table I:** Follow up of serum Her-2/neu at 3 months, 6 months & 1 yr (+2 wk) after treatment

Treatment (n) Serum	Serum Her-2/neu (baseline)	Serum Her-2/neu (post-treatment)
3 months (±2 wk) after treatment		
Surgery + CT (36)	32.47 ± 23.58	20.35 ± 14.4*
Surgery + RT (12)	28.08 ± 18.23	18.92 ± 6.47
6 months (±2 wk) after treatment		
Surgery + CT (9)	32.47 ± 17.79	20.3 ± 7.29*
Surgery + CT+tamoxifen (5)	28.08 ± 18.44	24.2 ± 3.94
Surgery + CT + RT (10)	52 ± 19.97	29.5 ± 13.74*
Surgery + CT+RT + tamoxifen (4)	25.7 ± 5.64	22.4 ± 5.2
12 months (±2 wk) after treatment		
Surgery + CT (6)	30.30 ± 11.68	12.79 ± 2.48*
Surgery + CT + tamoxifen (3)	33.43 ± 18.90	24.09 ± 7.92
Surgery + CT + RT (9)	53.90 ± 17.01	20.69 ± 8.7*
Surgery + CT + RT + tamoxifen (3)	44.86 ± 37.19	32.1 ± 20.72

Values are mean ± SD;

* *P*< 0.05 compared to baseline CT, chemotherapy; RT, radiotherapy

**Table II:** Comparison between Her-2/neu overexpression assayed by IHC and ELISA in patients with breast carcinoma (n=100)

IHC result (n=100)	Total no. of patietns	Serum Her-2/neu,<15ng/ml No. (%)	Serum Her-2/neu,>15ng/ml No. (%)
Negative (0/1)	71	55 (77.5)	15 (21.1)
Positive (2/3)	29	11 (37.9)	19 (65.5)
Total	100	66 (66.0)	34 (34.0)
Inference	IHC positivity was significantly associated with serum Her-2/neu,(>15 ng/ml) with sensitivity of 55.9% and with Mc Nemar *P*=0.424 and Kappa Co-efficient of agreement of k= 0.422 (Fair agreement)		

**Fig. 1:**
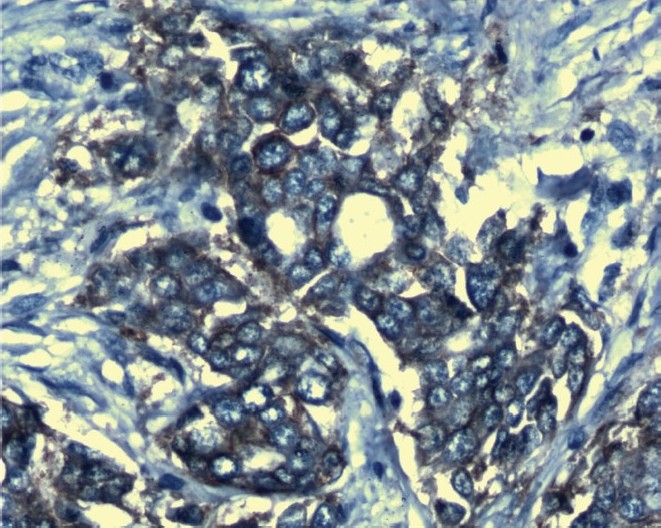
Her-2/neu positive slide stained with Haematoxylin (X 1000).

**Fig. 2:**
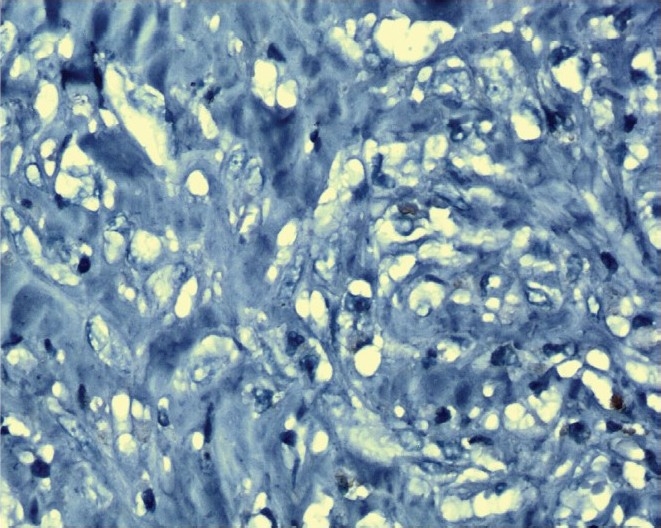
Her-2/neu negative slide stained with Haematoxylin (X 1000).

Immunohistochemical analysis using rabbit monoclonal antibody detected 29 cases with Her-2/neu overexpression (Fig. 1) and 71 cases were negative for Her-2/neu overexpression (Fig. 2). Baseline concentrations of serum Her-2/neu were elevated (>15 ng/ml) in 34 per cent of the patients and 66 per cent cases were Her-2/neu negative (Her-2/neu,<15ng/ml).

IHC positivity was significantly associated with serum Her-2/neu levels, >15 ng/ml with a sensitivity of 55.9 per cent (Mc Nemar P=0.424 and Kappa Co-efficient of agreement of k= 0.422).

## Discussion

Her-2/neu is overexpressed in both primary and metastatic breast cancer and predicts poor prognosis. Several investigators have suggested that Her-2 positivity of breast carcinomas may be indicative of resistance to hormonal (predominantly tamoxifen) therapy^13^–^15^. Her-2/neu overexpression is also associated with enhanced phosphorylation of both serine and tyrosine residues in the ER. Both alterations may be significant for ligand-independent ER activation with loss of the inhibitory effect of tamoxifen on ER-mediated transcription, providing a viable mechanism to explain the association of Her-2/neu with tamoxifen resistance^22^.

Our findings confirmed that Her-2/neu overexpression was associated with aggressive tumour features such as increased stage. Krainer *et al*^23^ have reported a significant correlation between serum concentrations of soluble Her-2/neu and tumour size or axillary lymph node involvement. Molina & coworkers^24^ showed that cleavage of the extracellular domain leads to increased phosphorylation of the intracellular tyrosine kinase. This observation suggests that circulating Her-2/neu level not only is a marker of tumour overexpression but also is indicative of the degree of receptor activation. Fehm & colleagues^25^, using a multivariate analysis, concluded that when serum Her-2/neu results were adjusted for tumour load with CA15-3, serum Her-2/neu remained an independent marker of tumour aggressiveness and reflected the biologic behaviour of the tumour.

In our study majority of the women were between the age group 40-55 yr. Pritchard *et al*^26^ showed that highest percentage (58.8%) of breast cancer belonged to 40-49 yr age group. We found a negative correlation between serum Her-2/neu and ER/PR expression. It has been hypothesised that Her-2 overexpression may interact with some of the metabolic pathways triggered by the activation of the ER. A possible explanation for the lower PR concentration in the presence of Her-2 overexpression is the activation of the P13K-Akt-mTor pathway by an increased growth factor activity. It has been shown that the activation of growth factor receptors such as Her-2/neu can result in direct phosphorylation and activation of ER in an estrogen-independent manner, which may itself be an important mechanism for tamoxifen resistance^27^.

By Lee *et al*^28^ demonstrated that serum Her-2/neu levels may serve to monitor neoadjuvant therapy in Her-2/neu positive breast cancer. Patients who were treated with surgery+CT+RT showed significantly (*P*<0.05) reduced serum Her-2/neu levels, showing good response to treatment. Patients who were treated with tamoxifen in addition to the above regimen did not show any significant response. Circulating Her-2/neu levels may be a better indicator of resistance to chemotherapy than the expression of Her-2 in the primary tumour^29^.

In this study, a statistically significant association between tissue Her-2/neu and serum Her-2/neu levels was also observed. In contrast to tissue testing which is a one time event, monitoring the circulating levels of the Her-2/neu in patients with breast cancer provides a real-time assessment of the Her-2/neu status which may be useful for managing the patients^29^.

In conclusion, the findings of this study support the hypothesis that overexpression of Her-2/neu is associated with tamoxifen unresponsiveness or a more aggressive phenotype of ER-positive breast cancer. Our study also revealed that serum Her-2/neu level was useful for predicting tissue Her-2/neu status and response to chemotherapy. Her-2/neu serum test should be performed more frequently in woman with breast cancer irrespective of their hormone receptor status to suggest modifications in systemic adjuvant therapy.
